# The sociodemographic patterning of drinking and binge drinking in Estonia, Latvia, Lithuania and Finland, 1994–2002

**DOI:** 10.1186/1471-2458-7-241

**Published:** 2007-09-13

**Authors:** Ville Helasoja, Eero Lahelma, Ritva Prättälä, Janina Petkeviciene, Iveta Pudule, Mare Tekkel

**Affiliations:** 1 National Public Health Institute, Department of Epidemiology and Health Promotion, Finland; 2 Department of Public Health, University of Helsinki, Finland; 3 Institute for Biomedical Research, Kaunas University of Medicine, Lithuania; 4 Health Promotion Centre, Latvia; 5 National Institute for Health Development, Department of Epidemiology and Biostatistics, Estonia

## Abstract

**Background:**

Despite the relatively low recorded alcohol consumption level, the Baltic countries (Estonia, Latvia and Lithuania) and neighbouring Finland suffer from similar harmful consequences related to the use of alcoholic beverages, including socio-economic inequalities in alcohol related mortality. Comparative evidence is needed to understand harmful drinking patterns and to implement preventive alcohol policies also in the Baltic countries. This study compared heavy and binge drinking by sex, age, education, urbanisation and marital status in the Baltic countries and Finland.

**Methods:**

The data were nationally representative postal surveys conducted in Estonia (n = 6271), Latvia (n = 6106), Lithuania (n = 7966) and Finland (n = 15764) during 1994–2002. The criterion for heavy drinking was at least 15 portions weekly among men, and at least five among women, and for binge drinking at least six portions per one occasion.

**Results:**

Heavy drinking was more common among younger participants in all countries, and in Latvia among the less-educated. Among Finnish men, and among women from all countries except Latvia, the better-educated were more often heavy drinkers. In Latvia and Finland, urban men, and in all countries, urban women, were more often heavy drinkers. Heavy drinking was more common among non-married Lithuanian and Finnish men, and Finnish women. Binge drinking was more common among less-educated Estonian and Latvian men, and among younger and less-educated women in all countries.

**Conclusion:**

Our results support the continued power of traditional drinking habits in the North Eastern part of Europe. In the future the target groups for prevention of excessive drinking should also include young and less-educated women in all four countries studied.

## Background

Despite the relatively low recorded alcohol consumption level [1,2], the Baltic countries (Estonia, Latvia and Lithuania) and Finland suffer from harmful consequences related to the heavy use of alcoholic beverages. This may be partly due to the undercoverage of consumption statistics, but people's drinking patterns [3] are likely to contribute as well. These countries are usually regarded as sharing a common European drinking pattern, which is particularly evident in the Northern European countries [4] and is characterised by high consumption at weekends. This pattern is known to contribute to harmful consequences of drinking, including socio-economic inequalities in premature mortality in Finland [5].

In the Baltic countries heavy alcohol use is recognised as a social problem [6], and there is aggregate level evidence on the adverse consequences of drinking and alcoholism [7,8]. In a comparative cross-sectional study in 1997, weekly alcohol consumption was associated with young age among both sexes, and with higher income among women [9]. In many Eastern and Western European countries, heavy drinking is patterned by key sociodemographic factors, such as age, sex, ethnicity, family status, socioeconomic status and living environment [10].

Little evidence is, however, available on changes in the sociodemographic patterning of heavy drinking in the 1990s and early 2000s in the Baltic countries, although mortality studies suggest that this will likely become an important issue in the near future [11-13]. Such evidence is needed to better understand these harmful drinking patterns and to efficiently implement preventive alcohol policies [1] as mere regulation of supply does not necessarily lead to diminished alcohol-related harm [14].

The Finbalt Health Monitor data [15] allow harmonised comparisons of alcohol drinking habits of the Baltic countries. Moreover, the data provide comparison to Finland, a neighbouring country that shares many long-term cultural and historical characteristics with the Baltic countries, but has particularly during the post second world war period been more close the Scandinavian and Western European countries. There is also evidence on rigorously-surveyed alcohol consumption habits and reliable consumption statistics from Finland, which thus provides both a methodological and substantial yardstick for interpretation of the comparative results from the Baltic countries.

This study aims to compare the patterning of heavy drinking and binge drinking by key sociodemographic determinants, i.e. sex, age, education, urbanisation, marital status and ethnicity in Estonia, Latvia, Lithuania and Finland in 1994–2002. More specifically, the aims are to examine:

1) What is the overall sociodemographic patterning of heavy drinking in these four countries from 1994–2002?

2) What kind of changes occurred from 1994–2002 in the sociodemographic patterning of heavy drinking in these four countries?

3) What is the overall sociodemographic patterning of binge drinking in these four countries from 2000–2002?

## Methods

Data were collected from nationally representative cross-sectional postal surveys from the Finbalt Health Monitor project in 1994, 1996, 1998, 2000 and 2002 in Estonia, Lithuania and Finland. Lithuanian data on weekly alcohol consumption has been available since 1996. In Latvia, surveys were carried out since 1998. The national surveys have been accepted by respective ethics committees. In Estonia by the Tallinn Medical Research Ethics Committee, in Lithuania by the Lithuanian Bioethics Committtee, in Latvia the Ethical commitee of Riga Stradins university and in Finland by the ethical Committee of the Public Health Institute. In all the countries the covering letter of the questionnaire is formulated in a way that a respondent provides an informed consent by returning the questionnaire.

The methodology and questionnaires of the surveys have been standardised [15]. The data have also been compared to census information [16]. Generally, the youngest age group and men are slightly underrepresented but the proportion of urban and non-native population corresponds well with censuses. Despite lack of comparable information, we can assume that those with better education and married are somewhat overrepresented. Late response, as well as unit- and item- non response have also been analysed. The direction of bias is likely to be similar in all four countries.

In Estonia, Lithuania and Finland, the questionnaire measured education as the total number of years of education. In Latvia, the questionnaire measured education according to four standard educational levels. In all four countries, the level of urbanisation was based on the administrative classification of the respondent's address. Ethnic origin was dichotomized as 'natives' and 'non-natives'. In the Baltic countries, 'non-natives' comprised mainly Russians. (Additional File 1)

Weekly alcohol consumption was measured with the following question: 'How many glasses (regular restaurant portions) or bottles of the following drinks have you consumed during the last week (7 days)? If you have not had any, mark 0, i. medium strong or strong beer __ bottles, ii. free-mixed highballs __ bottles, iii. strong alcohol, spirits __ restaurant portions (4 cl.), iv. wine or equivalent __ glasses. In Finland an additional beverage, 'cider or other light wine', was included with 'wine' in our analysis. In Lithuania, the consumption of 'free-mixed highballs' was not asked. Empty responses were included in the 0 consumption category. Distribution of weekly alcohol consumption was skewed to the right in all countries (skewness 4.95–9.03), indicating that the proportion of non-drinkers and moderate drinkers was substantial.

Because of this type of data we decided to categorize our respondents into high and low alcohol consumption groups by using comparable criteria for all the countries involved. The feasible cutt-off point was found to be at least 15 portions per week among men, and at least five portions per week among women. It is inevitable that higher consumption group may contain both heavy occasional and moderate frequent consumption, however, in this paper it is called heavy drinkers. (Additional File 2)

There is no general consensus on the exact limits of moderate consumption or binge drinking and various cut off points as grams of alcohol [17] or qualitatative characteristics of drinking occasions [18] have been suggested. A simple measure of consuming five or six drinks per occasion has been related to increased risk of mortality [5]. Also Finbalt surveys of the years 2000 and 2002 have asked binge drinking with the following question: 'How often do you drink at least six (regular restaurant) portions of alcohol per one occasion? 1) never, 2) less than once a month, 3) at least once a month, 4) at least once a week, 5) daily or almost daily. In all countries mean weekly consumption was higher among those who more often drank six or more portions. Asymptotic significance for difference in the Kruskall-Wallis test was *P *< 0.001 among men and women in all countries. In this study the criterion for binge drinking was six or more portions at least once a week among men, and at least once a month among women (Additional File 2).

We estimated the consumption of pure alcohol in our data assuming that each reported portion equals one medium-size bottle (0.33) of medium-strong beer (alcohol content 4.2%). Overall estimates for mean annual pure alcohol consumption in the study period were 2.7 litres per capita in Estonia (years 1994–2002), 2.9 in Latvia (years 1998–2002), 2.7 in Lithuania (years 1996–2002) and 4.4 in Finland (years 1994–2002). Compared to official consumption statistics [2,8,19-22] from the nearest respective year, the annual coverage ratio (the proportion that our year specific annual estimate covers of consumption statistic figure) as percentages varied between 25–111% in Estonia, 36–47% in Latvia, 25–31% in Lithuania and 54–70% in Finland. The substantial variation in these ratios, as can be seen especially in Estonia, is mainly due to published consumption figures being different depending on the source.

The analysis was carried out in three phases. In the first phase, we examined sociodemographic differences in heavy drinking cross-sectionally in order to include all relevant factors with sufficient detail and statistical power. Logistic regression analysis was used; all models were fitted separately for each country among men and women, and the main effects were included in their assumed temporal order. The effect of age was added first, followed by education, urbanisation and marital status. Interactions between education and each sociodemographic factor were included separately into the mutually adjusted main effects model. (Additional files 3 and 4)

The statistical significance of these effects was assessed with scaled deviance and change in the degrees of freedom (Δ SD and Δ DF), and the criterion for a statistically significant effect was *P *< 0.05. *P*-values for the main effects appear in Additional files 3, 4, 5, 6, and in text for interactions.

In the second phase, we examined the changes over time in the sociodemographic differences in heavy drinking. Again logistic regression analysis was used. The effect of study year was added first, followed by age (dichotomous: 20–42, 43–64 years), education, urbanisation (dichotomous cities + capital, towns + villages) and marital status (dichotomous). Models consisting of each explanatory variable were also examined. We assessed changes over time in the associations by including interactions between study year and each sociodemographic factor separately into the mutually adjusted main effects model. The differences between study years are presented in Additional files 3 and 4.

In the third phase, binge drinking was examined with logistic regression analysis. The effect of age was added first, followed by education, urbanisation and marital status. Interactions between education and each sociodemographic factor were included separately into the mutually adjusted main effects model. (Additional files 5 and 6)

In Estonia and Latvia, we also analysed the differences in heavy drinking and binge drinking between the natives and non-natives. The effect of ethnicity was added first, followed by age, education, urbanisation and marital status. Finally interactions between ethnicity and each of the other factors were added separately into the mutually adjusted main effects model. Urbanisation was excluded from the models of binge drinking because of the limited number of cases. Statistically significant differences between the ethnic groups appear in the text.

## Results

### Changes over time in heavy drinking

Heavy drinking was more common in Finland than in the Baltic countries among both men and women (Additional File 2). In Lithuanian men heavy drinking increased compared to the reference year 1996 (Additional File 3). Similarly, heavy drinking increased among women (Additional File 4), peaking in 2000 in Estonia, in 2002 in Lithuania, and in 1998 in Finland. Among Finnish men, there was interaction between study year and education (*P *< 0.01), suggesting that the educational gradient was steepest in 1998. No consistent change occurred, however, in the gradient over time (data not shown).

### Sociodemographic differences in heavy drinking

Additional files 3 and 4 show that mutual adjustment did not substantially affect the sociodemographic differences in heavy drinking, and conclusions from both unadjusted and adjusted models in all countries were similar. Heavy drinking was more common among the younger than among older respondents of both genders in all countries. In Latvian men, heavy drinking was more common among the less-educated than among the higher-educated. In contrast, heavy drinking was more common among the better-educated Finnish men. In Lithuanian and Estonian men, heavy drinking did not vary by educational level. Heavy drinking was more common among better-educated women in all countries, except for Latvia, where it occurred more frequently among less-educated women. The effect of education was statistically significant in Finnish men, and in women in all countries studied. Heavy drinking was more common among urban Latvian and Finnish men, and in urban women of all the other countries except Lithuania. Heavy drinking was more common among divorced Lithuanian men, as well as among non-married Finnish men and women.

The educational gradient was steepest among Finnish men and women aged 49–64 years, and the interaction of education and age was statistically significant (*P *< 0.001). In younger age groups educational differences were less marked. In Latvian women, the educational gradient was steepest in the age group of 34–39 years. The educational gradient was, however, basically similar to that presented in Additional File 4 in all age groups even though the interaction of education and age was statistically significant (*P *< 0.05). (data not shown)

Heavy drinking was more common among the non-natives than the native Estonian women. The mutually adjusted odds ratio was 1.40 (1.07–1.83), and the effect of ethnicity was statistically significant (*P *< 0.001). In Latvian men, the age gradient was steeper among non-natives than among natives, and the interaction between ethnicity and age was statistically significant (*P *< 0.05). In Estonian men, the educational gradient was observed only among non-natives, where heavy drinking was most common among the low-educated. This educational difference in non-native Estonian men was statistically non-significant but the statistical significance of interaction between ethnicity and education was *P *< 0.05. (data not shown)

### Sociodemographic differences in binge drinking

Additional files 5 and 6 show that mutual adjustment did not substantially affect the sociodemographic patterning of binge drinking, but the educational gradient became somewhat steeper among women. Binge drinking was more common among younger women than among their older counterparts in all countries, but we found no consistent age gradient among men. Binge drinking was more common among the less-educated Estonian and Latvian men, and among the less-educated women in all countries.

In Estonian men, however, the difference between the low-educated and the higher-educated was statistically non-significant. The effect of education was statistically significant among Estonian men and Latvian men and women, and among Finnish women. Binge drinking was more common among urban Finnish men and women, and among Latvian women than among their less urban counterparts. In Estonian men and in Finnish men and women, binge drinking was more common among the non-married than among the married.

None of the interactions between education and other independent variables was statistically significant in the countries studied. Binge drinking, when mutually adjusted for, was more common among non-native Estonian women (OR 1.84, 1.23.-2.76) than among natives.

## Discussion

We found that the sociodemographic patterning of heavy drinking was more consistent among women than among men. Heavy drinking was also more common among younger, better-educated and urban men and women in Estonia, Lithuania and Finland. There were no major changes over time in this patterning. Binge drinking was more common among less-educated Estonian and Latvian men and among younger and less-educated women in all countries. In Latvia, both heavy drinking and binge drinking were more common among the less-educated.

According to the estimated coverage rates of this study, underrreporting was likely to be substantial -particularly in the Baltic countries. Research into heavy drinking is compromised when alcohol consumption of the majority of respondents falls within the limits of moderate drinking [23] in populations with traditionally irregular drinking patterns [24]. However, the portions that people actually drink may be larger than in our total consumption estimates [25]. In addition, our data showed a consistent association between the frequency of binge drinking and mean weekly consumption which suggests that, despite underreporting, the data can serve to identify subgroups where adverse drinking habits and problems are most likely to exist [26].

It has been argued that in the Baltic countries lay people may respond to alcohol surveys in a way they expect the authorities would like them to do [27-29]. Another study indicating considerably higher underreporting of alcohol consumption in Russian Karelia than in Finnish Karelia partly supports this [30]. As our postal survey resulted in a higher prevalence of weekly consumption of alcohol, especially among women (Estonia 52% vs. 26%, Latvia 57% vs. 8%, Lithuania 65% vs. 14%), than did another study using personal face to face interviews [9], a social undesirability effect leading to underreporting may be real.

Sociodemographic differences were more pronounced among women, especially with regard to binge drinking. The use of six portions per one occasion may better indicate excessive use of alcohol among women as studies suggest that simple questions on alcohol consumption are likely to underestimate gender differences in the prevalence of heavier drinking [31]. However, consuming six portions per one occasion has been associated with increased mortality among men in Finland [5].

As with other comparative studies [9], age was the most consistent determinant across all countries studied and in both genders. In men, however, there were no consistent age differences in binge drinking, possibly indicating that this traditional habit remains popular among all male cohorts. Moreover, other studies [32] support the consistent age gradient in binge drinking among women, where young women are more often binge drinkers, which may suggest an emerging trend in drinking habits. The data on time-trends for binge drinking cover a short period only, but the increase over time in heavy drinking among women in all countries studied except Latvia suggests change.

The Finnish experience shows that drinking habits may change within a relatively short period of time [33]. We also found that in Finland, education was a more important determinant of heavy drinking in older age groups, but no interaction between age and education for binge drinking existed. This may be due to a tradition of binge drinking in Finland, but younger people in all educational groups are adopting 'new' alcohol consumption habits.

The educational gradient was consistent among men in Latvia, and to some extent in Estonia: the less-educated were more often heavier drinkers. This is in accordance with other surveys [34,35] and follows the general message of mortality studies, which suggests that excessive alcohol consumption is more common among the less-educated [11,13]. The educational gradient among women in Estonia, Lithuania and Finland showed that while the better-educated were more likely to be heavy drinkers, the less-educated were more often binge drinkers, which follows the typical European drinking pattern particularly common in Northern Europe [4]. In Latvian women, however, the pattern resembled that of Latvian men, with the less-educated drinking more according to both measures.

Explanations for excessive drinking among the less-educated in Latvia include low price as well as illegal production and sale of alcohol [32]. However, these phenomena also exist in the other countries studied. One partial explanation for the consistent educational gradient among Latvian men only might be that the response rates were highest in Latvia and the difference between the respondents and non-respondents was probably smaller than in other countries studied. Previous surveys measuring weekly alcohol consumption among Baltic men [9], however, suggest less marked educational differences.

In general, urban people in all four countries tended to drink more. In Finland in particular, the differences were consistent, which agrees with previous evidence [36]. As other studies [9,37] have indicated, among Baltic men urban-rural differences in drinking were inconsistent. Nevertheless, the more common drinking among urban women may reflect modern urban life styles.

The association of marital status with drinking was generally expected, as drinking is more common among the non-married. This was the case in Finland especially, but the association was less consistent in the Baltic countries, partly due to low statistical power of the analyses. Our data provided slight support, however, to the assumption that non-married-, and particularly divorced men are a risk group in the Baltic transition economies. For women, smaller-than-expected differences between marital status groups in the Baltic countries may be partly due to parenthood, which has been associated with women's drinking elsewhere [38] but could not be taken into account.

In Estonia non-native women drank more often than did the natives, which partly contrasts with an earlier survey [9], but agrees with ethnic differences in mortality due to alcohol [39]. For Estonian men a steeper educational gradient in heavy drinking existed among the non-natives, which agrees with the educational gradient found in alcohol-related mortality [40]. A previous survey has also found a similar lack of ethnic differences in Latvia [9].

## Conclusion

We found no major changes over time in the sociodemographic patterning of heavy drinking, which may suggest that the years 1994–2002 were a period of relative stabilisation in the re-independent Baltic countries after the major shock effects of the economic transition after the collapse of the Soviet regime in the early 1990s. The possible contribution of drinking habits to the increasing educational differences in mortality [13] and the age and educational gradients found in this study, however, call for further research on binge drinking with longer time trends.

In short, our study on Estonia, Latvia, Lithuania and Finland supports the continued power of traditional drinking habits in this part of North Eastern Europe. Thus heavy drinking and binge drinking is still much more common among men than women. However, in the future young and less-educated women should be included as a new target group for prevention of excessive drinking in all four countries studied.

## Competing interests

The author(s) declare that they have no competing interests.

## Authors' contributions

All authors participated in the design of the study and writing the manuscript. VH performed the statistical analysis and drafted the first versions. All authors have read and approved the final draft of the paper.

## Pre-publication history

The pre-publication history for this paper can be accessed here:



## Supplementary Material

Additional file 1 Basic characteristics of the study material. Click here for file

Additional file 2 Consumption of alcohol beverages in 1994–2002 (Latvia 1998–2002, Lithuania 1996–2002). Click here for file

Additional file 3 Heavy drinking: Consumption of at least 15 alcohol portions per weekamong men by sociodemographic variables in 1994 (excl. Lithuania), 1996, 1998, 2000 and 2002. Unadjusted (unadj.) and mutually adjusted for all other terms in the model (adj) odds ratios (OR) and their 95% confidence intervals (CI). Click here for file

Additional file 4 Heavy drinking: Consumption of at least 5 alcohol portions per weekamong women by sociodemographic variables in 1994 (excl. Lithuania), 1996, 1998, 2000 and 2002. Unadjusted (unadj.) and mutually adjusted for all other terms in the model (adj) odds ratios (OR) and their 95% confidence intervals (CI). Click here for file

Additional file 5 Binge drinking among men (at least once a week) by sociodemographicvariables in 2000 and 2002. Unadjusted (unadj.) and mutually adjusted for all other terms in the model (adj) odds ratios (OR) and their 95% confidence intervals (CI). Click here for file

Additional file 6 Binge drinking among women (at least once a month) bysociodemographic variables in 2000 and 2002. Unadjusted (unadj.) and mutually adjusted for all other terms in the model (adj) odds ratios (OR) and their 95% confidence intervals (CI). Click here for file
